# Cross-Species Amyloid-like Features Shared by Mammalian and *Clostridioides difficile* Proteins

**DOI:** 10.3390/microorganisms14040821

**Published:** 2026-04-02

**Authors:** Weichen Gong

**Affiliations:** 1 Laboratory of Animal Food Function, Graduate School of Agricultural Science, Tohoku University, Sendai 980-8572, Japan; gong.weichen.c1@tohoku.ac.jp; 2 Livestock Immunology Unit, International Education and Research Center for Food and Agricultural Immunology (CFAI), Graduate School of Agricultural Science, Tohoku University, Sendai 980-8577, Japan

**Keywords:** amyloid protein, amyloidogenic motifs, serum amyloid A

## Abstract

Functional amyloids are widely distributed in bacteria and play important roles in biofilm formation and microbial physiology. However, most currently known bacterial amyloids have been identified through sequence homology to a limited number of prototype proteins, such as the curli subunit CsgA of *Escherichia coli*. This approach may overlook amyloidogenic sequences that lack recognizable similarity to these canonical systems. In this study, a cross-species, motif-based computational strategy was used to explore whether conserved sequence features derived from mammalian serum amyloid A (SAA) proteins could provide clues for identifying potential amyloidogenic motifs in bacterial proteomes. Comparative analysis of mammalian SAA isoforms identified a conserved sequence segment with predicted aggregation propensity, within which the hydrophobic motif SIAIILCILIL was observed in murine SAA3. Database searches revealed that similar sequence motifs occur in several proteins encoded by Gram-positive bacteria, including multiple proteins in *Clostridioides difficile*. To further explore whether *C. difficile* produces extracellular structures capable of interacting with amyloid-binding dyes, Congo Red-supplemented agar assays were performed. After 48 h of growth, both clinical isolates and a laboratory reference strain exhibited Congo Red-binding colony phenotypes. Because Congo Red binding can arise from several extracellular components and cannot be attributed to a specific protein or sequence motif, these observations should be interpreted cautiously. Taken together, this study presents a motif-based computational framework for identifying candidate amyloidogenic motifs across species and highlights sequence features in bacterial proteomes that may warrant further biochemical and structural investigation. The results should be regarded as hypothesis-generating and provide a basis for future experimental validation of potential amyloid-forming proteins in bacteria.

## 1. Introduction

Amyloid proteins are a diverse class of polypeptides with a strong tendency to aggregate into insoluble fibrils, a process often associated with disease [1]. Pathological amyloid deposition occurs in a wide range of mammalian disorders. For example, amyloid-β accumulation is a hallmark of Alzheimer’s disease, α-synuclein fibrils are linked to Parkinson’s disease, prion protein aggregates cause spongiform encephalopathies, and serum amyloid A (SAA) fibrils are causally associated with systemic AA amyloidosis [2,3,4,5,6]. These examples underscore the clinical importance of amyloid proteins and the need to better understand their structural determinants and biological functions.

In recent years, the concept of “functional amyloids” has emerged, particularly in bacteria. Several microorganisms produce extracellular amyloid proteins that support biofilm formation and stability. The archetypal example is the curli system of *Escherichia coli*, where the major subunit CsgA polymerizes into β-sheet-rich fibrils forming the structural backbone of the biofilm matrix [7,8]. Furthermore, *Bacillus subtilis* produces TasA, which assembles into fibers essential for biofilm integrity [9]. *Pseudomonas* species secrete Fap proteins, such as FapC, that assemble into β-solenoid fibrils, whereas *Staphylococcus aureus* synthesizes phenol-soluble modulins (PSMs), short amphipathic peptides that can adopt amyloid-like conformations and contribute to virulence and host interactions [10,11,12]. Collectively, these studies demonstrate that bacterial amyloid or amyloid-like proteins are closely associated with biofilm formation and stability. Biofilms represent a dominant bacterial lifestyle and confer numerous advantages, including enhanced resistance to environmental stresses, antimicrobial agents, and host immune defenses, thereby promoting bacterial persistence and pathogenicity [13]. Notably, bacterial biofilms are not composed exclusively of microbe-derived components. Accumulating evidence indicates that host-derived amyloidogenic proteins can also be incorporated into biofilm matrices. Among these, SAA is one of the most extensively studied host amyloid proteins detected in infection-associated biofilms [14,15]. Despite the coexistence of bacterial and host amyloid or amyloid-like proteins within biofilms, potential cross-species interactions between these components remain largely unexplored. In parallel, recent structural and mechanistic studies of bacterial amyloids have provided valuable insights into their assembly pathways, polymorphisms, and functional versatility.

Despite this progress, the bacterial amyloids characterized to date are dominated by β-sheet architectures, reflecting the canonical hallmark features of amyloid fibrils [16]. In contrast, many mammalian amyloid precursors retain substantial α-helical content in their soluble states [17,18]. A representative example is SAA, a highly conserved acute-phase protein widely expressed in mammals. Beyond serving as a biomarker of inflammation, SAA participates in lipid transport through its association with high-density lipoproteins (HDL), modulates innate immune responses via interactions with Toll-like receptors and formyl peptide receptors, and acts as a chemoattractant for immune cells [19,20,21]. Structural studies have shown that soluble SAA adopts a four-helix bundle, and comparative sequence analyses indicate that several α-helical regions are strongly conserved across species [22]. However, the biological significance of these conserved α-helical motifs remains unsolved.

Importantly, amyloid formation has traditionally been associated with intrinsically β-sheet-prone sequences. Consequently, most computational prediction tools and discovery pipelines are optimized to identify regions with inherent β-aggregation propensity. However, accumulating structural and biochemical evidence indicates that certain α-helical proteins can undergo α-helix-to-β-sheet conformational transitions, ultimately forming cross-β amyloid architectures [23]. In these cases, amyloidogenic potential does not necessarily arise from pre-existing β-rich sequences but from structural plasticity and context-dependent secondary structure rearrangement.

Although both bacterial and mammalian amyloids ultimately adopt cross-β architectures in their fibrillar states, many well-characterized bacterial amyloids have been identified based on intrinsically β-prone sequence features that predispose them to direct β-aggregation. In contrast, several mammalian amyloid precursor proteins contain substantial α-helical or structurally flexible regions in their soluble forms, which subsequently undergo conformational rearrangement during amyloid formation. This distinction highlights differences in discovery paradigms rather than final fibrillar architecture [23,24,25].

This contrast raises an intriguing and unexplored question: can conserved elements in mammalian amyloid proteins, such as SAA, provide predictive clues for identifying novel bacterial amyloids? While bacterial functional amyloids are largely β-sheet-based, the existence of conserved α-helix motifs in mammalian amyloids suggests potential cross-kingdom structural principles or sequence signatures that remain to be defined. Up till now, systematic computational approaches to identify such helix-derived aggregation-prone motifs remain limited. Most reported examples have been discovered incidentally rather than through hypothesis-driven screening strategies. Addressing this gap may not only improve our ability to predict and characterize bacterial amyloid proteins, but also illuminate the evolutionary and functional relationships between host and microbial amyloids. Here, we introduce a cross-species, motif-based analytical framework designed to capture conserved sequence motifs that may potentially contribute to amyloid-like aggregation. By focusing not solely on static β-propensity but on evolutionarily conserved sequence features associated with structural adaptability, this approach expands the conceptual and methodological landscape of amyloid discovery beyond traditional β-centric paradigms.

Therefore, the aim of this study was to investigate whether conserved sequence features derived from mammalian SAA proteins could be used to identify candidate amyloidogenic motifs in bacterial proteomes.

## 2. Materials and Methods

### 2.1. Overall Analytical Workflow

The overall analytical workflow of this study consisted of four main steps: (i) retrieval of mammalian SAA sequences and structural modeling, (ii) identification of conserved sequence motifs through multiple sequence alignment, (iii) prediction of aggregation propensity using PASTA 2.0, and (iv) database searches to identify homologous motifs in bacterial proteomes. These steps were performed sequentially using publicly available tools with default parameters unless otherwise specified. No manual filtering or subjective selection criteria were applied beyond the parameters described above.

### 2.2. Sequence Retrieval and Structural Modeling

All serum amyloid A (SAA) sequences were retrieved from the UniProt database (https://www.uniprot.org/, accessed on 10 February 2026). The sequences were subsequently submitted to the SWISS-MODEL server (https://swissmodel.expasy.org/, accessed on 10 February 2026) to generate homology-based structural models. Modeling was performed in automated mode using the server’s default parameters. Template identification was carried out automatically through BLAST (NCBI BLAST+ suite) and HHblits (HH-suite v3.3.0) searches against the SWISS-MODEL template library (SMTL), derived from the Protein Data Bank (PDB). Templates were ranked based on sequence identity, alignment coverage, and the Global Model Quality Estimation (GMQE) score. The top-ranked template suggested by the server was selected without manual intervention. Model quality was further assessed using the QMEAN scoring function provided by SWISS-MODEL, and only models with acceptable quality estimates were retained for subsequent structural analyses. No additional manual refinement or energy minimization was performed.

To ensure reproducibility, the modeling procedure was independently repeated three times for each sequence, and consistent template selection and model quality metrics were obtained across runs.

### 2.3. Sequence Alignment and Similarity-Based Clustering

To investigate sequence conservation, all the SAA sequences were aligned using Clustal Omega (vesion 1.2.4), a multiple-sequence alignment tool (https://www.ebi.ac.uk/jdispatcher/msa/clustalo, accessed on 11 February 2026) [26]. Multiple-sequence alignment was performed using default parameters, without manual adjustment. The default settings included the use of the HHalign package (HH-suite v3.3.0) for profile-profile alignment, automatic guide tree construction, and standard gap opening and extension penalties as implemented by the server. No custom substitution matrices or iteration parameters were applied. The alignment output was examined to identify secondary structural elements, particularly α-helices. Comparative analysis revealed a conserved 19–amino acid α-helical segment across species. Similarity-based clustering was inferred from the aligned sequences.

Similarity-based clustering was explored based on the guide tree generated automatically by Clustal Omega (implemented at the EMBL-EBI server, accessed on 13 February 2026) during multiple-sequence alignment. Clustal Omega constructs this tree using pairwise sequence distances and mBed clustering to guide alignment order. No additional phylogenetic reconstruction methods (e.g., neighbour-joining or maximum likelihood) were applied, and bootstrap analysis was not performed.

To ensure reproducibility, the modeling procedure was independently repeated three times for each sequence, and consistent template selection and model quality metrics were obtained across runs.

### 2.4. In Silico Aggregation Propensity Prediction

The aggregation-related properties of the SAA sequences were evaluated using PASTA 2.0, an improved server for protein aggregation prediction (http://old.protein.bio.unipd.it/pasta2/, accessed on 14 February 2026). All analyses were performed using the default server parameters without manual modification. PASTA 2.0 predicts amyloidogenic regions based on pairwise β-sheet interaction energies calculated using a statistical potential derived from known protein structures. The default sliding window approach implemented by the server was applied, and no custom energy thresholds or window size adjustments were introduced. Amyloid propensity scores and aggregation-prone regions were identified according to the server’s internal scoring system.

Six parameters were extracted for each sequence: amyloid propensity, best energy, percentage of disorder, α-helical content, β-strand content, and coil content. Data were normalized and visualized in Python (version 3.9) using the pandas and matplotlib packages, and radar plots were constructed to compare aggregation propensities across sequences.

To ensure reproducibility, the modeling procedure was independently repeated three times for each sequence, and consistent template selection and model quality metrics were obtained across runs.

### 2.5. Database Search for Bacterial Homologs

Following the identification of a strongly aggregation-prone segment (SIAIILCILIL) within SAA, protein–protein similarity searches were performed using the NCBI protein BLAST (pBLAST) tool implemented in the NCBI BLAST suite (https://blast.ncbi.nlm.nih.gov, accessed on 14 February 2026). Searches were run against the ClusteredNR database using the algorithm set to blastp implemented in the NCBI BLAST suite (accessed on 15 February 2026). Organism filters were applied to target Gram-positive bacteria (taxid: 1239), Gram-negative bacterium 0471 (taxid: 204774), and *Clostridioides difficile* (taxid: 1496).

All searches were conducted using default parameters unless otherwise specified. The E-value cutoff was set to 0.05, with a word size of 5 and a maximum of 100 target sequences returned. The BLOSUM62 substitution matrix was used, with gap costs set to existence 11 and extension 1. Conditional compositional score matrix adjustment was enabled as implemented by the BLAST server. No additional percent identity or alignment coverage thresholds were imposed beyond the default BLAST ranking criteria. Low-complexity filtering was not enabled. Hits were ranked according to E-value and alignment score as provided by the BLAST output.

To ensure reproducibility, the modeling procedure was independently repeated three times for each sequence, and consistent template selection and model quality metrics were obtained across runs.

### 2.6. Bacterial Strains and Culture Conditions

Five *C. difficile* strains were used in this study. Strains#1, #2, #3, and #4 were isolated from a clinical bacterial mixture (a laboratory bacterial stock), and ATCC 9689 (*Clostridioides difficile* ATCC 9689) served as a reference. The reference strain was obtained from the American Type Culture Collection (ATCC). All strains were cultured in GAM liquid medium (Code #05422, Nissui, Tokyo, Japan) under anaerobic conditions generated using an AnaeroPack-Anaero (MITSUBISHI GAS CHEMICAL Co. Inc., Tokyo, Japan) at 37 °C.

### 2.7. Congo Red Binding Assay

To assess amyloid-like properties, Congo Red-supplemented GAM plates were prepared by adding 1% (*w*/*v*) Congo Red solution (Lot# 250502, Muto Pure Chemicals Co., Ltd., Tokyo, Japan) to the medium at a final dilution of 1:250, corresponding to a final Congo Red concentration of 40 µg/mL (0.004% *w*/*v*). Bacterial inoculations were directly streaked from stock cultures onto Congo RedGAM plates and incubated anaerobically for 48 h. Colonies exhibiting a red phenotype were considered positive for Congo Red binding.

### 2.8. Data Visualization and Figure Preparation

All figures were assembled using raw images obtained from the respective software platforms or databases. Final layouts and minor adjustments were made using Microsoft PowerPoint (version 16.100.2).

## 3. Results

To systematically investigate cross-species amyloid-like features, the Results are organized into four key aspects: (i) identification of conserved motifs in mammalian SAA, (ii) evaluation of aggregation propensity, (iii) detection of homologous motifs in bacterial proteins, and (iv) experimental assessment of Congo Red-binding phenotypes.

### 3.1. Conserved α-Helical Motifs in Mammalian SAA Proteins

**Figure 1:**
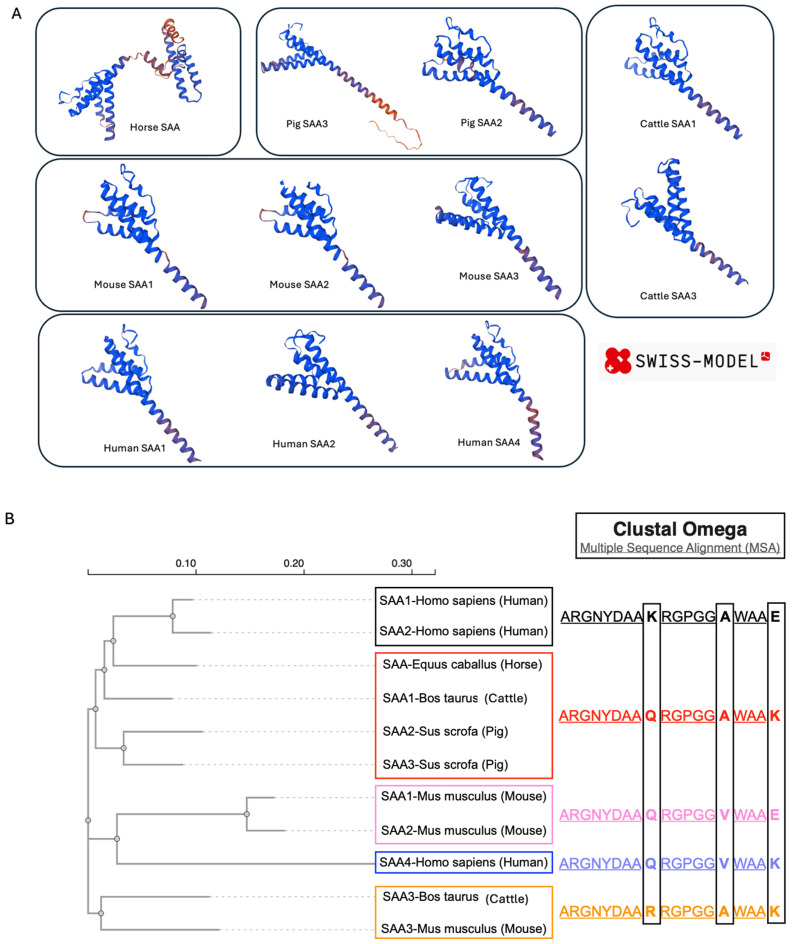
Structural modeling and sequence conservation of mammalian SAA isoforms (**A**) Predicted protein structures of representative mammalian SAA isoforms generated using SWISS-MODEL. (**B**) Multiple sequence alignment of SAA isoforms performed with Clustal Omega (implemented at the EMBL-EBI server) revealing a conserved 19–amino acid motif. Residue variations are observed at positions 9, 15, and 19, corresponding to point substitutions among species.

To investigate the structural features of mammalian SAA, I compared representative SAA sequences from several species, including horse (one isoform), pig (two isoforms), cattle (two isoforms), mouse (three isoforms), and human (three isoforms) (Table 1). Structural modeling using SWISS-MODEL predicted that all SAA monomers are predominantly α-helical (Figure 1A) [27]. Sequence alignment with Clustal Omega (implemented at the EMBL-EBI server) revealed a 19-amino acid segment highly conserved across species [26]. Three polymorphic sites were identified in this region: positions 9 (K/Q/R), 15 (A/V), and 19 (E/K). Based on the guide tree generated during multiple sequence alignment, human SAA1 and SAA2 cluster closely, as do mouse SAA1 and SAA2, whereas horse SAA, bovine SAA1, porcine SAA2/3, and murine SAA3 clustered into distinct subgroups. Human SAA4 formed a separate clade (Figure 1B).

These observations indicate that, despite sequence divergence, SAA proteins share conserved α-helical motifs, suggesting their potential functional significance. To further explore this possibility, I examined whether structural variability among isoforms correlates with their aggregation propensity.

### 3.2. Aggregation Propensity Analysis Identifies Murine SAA3 as Highly Amyloidogenic

Given the well-documented aggregative properties of SAA, I examined whether different host-derived SAAs vary in their aggregation propensities. Aggregation propensity was evaluated using PASTA 2.0, a physics-based algorithm that estimates inter-strand β-sheet pairing energies within a cross-β structural model [28]. Because amyloid fibrils are structurally defined by stabilized β-sheet stacking, an energy-based pairing framework provides mechanistic interpretability beyond simple sequence heuristics. In contrast to purely score-based or machine-learning-derived predictors, PASTA 2.0 outputs quantitative energy values that allow direct comparison of aggregation strength across species. Although the algorithm is primarily optimized for β-driven aggregation, it remains informative for assessing sequences that may undergo α-helix-to-β-sheet conformational transition during amyloidogenesis. It should be noted that PASTA 2.0 predicts aggregation propensity based on β-sheet pairing energies rather than the native secondary structure of the folded protein. Using in silico analysis with PASTA 2.0, I evaluated five parameters: best energy, percentage of disorder, α-helix content, β-sheet content, and coil content [28]. The analysis predicted that murine SAA3 had the highest aggregation potential, whereas human SAA1 had the lowest (Table 2). Comparative sequence analysis revealed that the SIAIILCILIL motif is uniquely present in the murine SAA3 sequence, suggesting that it may represent a candidate aggregation-prone motif. To assess its contribution to aggregation, I compared the predicted aggregation propensities of the isolated motif, the full-length murine SAA3 protein, and a truncated SAA3 sequence lacking the motif. In the truncated construct, the SIAIILCILIL segment together with its immediately adjacent N-terminal residues (MKP) was removed to avoid partial motif retention at the sequence boundary. The removal of SIAIILCILIL significantly reduced the predicted aggregation propensity of murine SAA3, suggesting that this motif contributes to the aggregation propensity predicted for murine SAA3 in the computational analysis (Table 3).

Having identified an amyloidogenic motif in murine SAA3, I subsequently investigated whether similar sequence motifs occur in microbial proteins, particularly in bacteria, where amyloids have been implicated in biofilm formation and pathogenicity.

### 3.3. Identification of SAA-Derived Amyloidogenic Motifs in Bacterial Proteins

**Figure 2:**
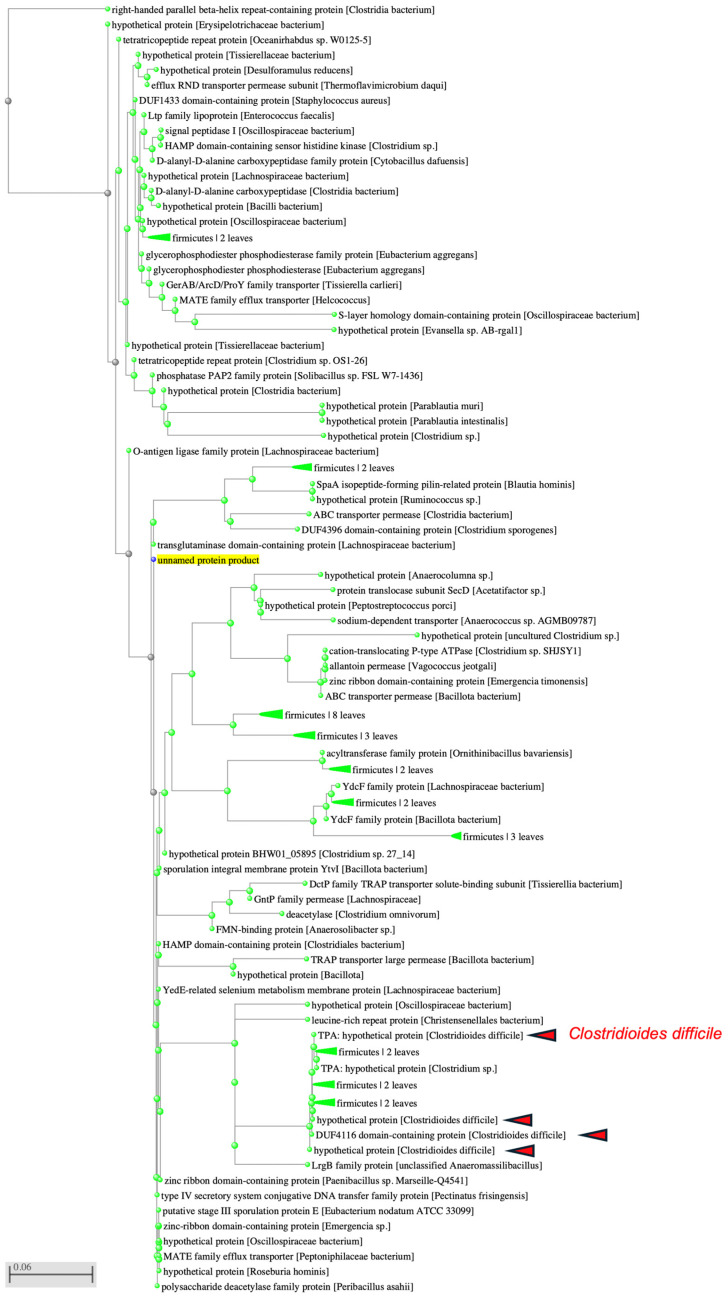
Identification of bacterial homologues of the SIAIILCILIL motif. pBLAST search (implemented in the NCBI BLAST suite) against Gram-positive bacteria revealed four homologous proteins in *Clostridioides difficile* containing the SIAIILCILIL motif: a TPA hypothetical protein, a DUF4116 domain–containing protein, and two additional hypothetical proteins.

**Figure 3:**
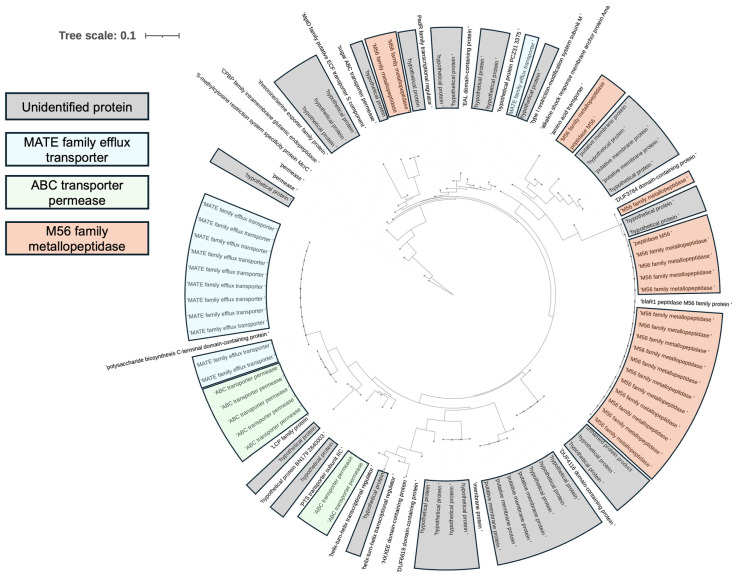
A broader pBLAST search within *C. difficile* (taxid: 1496) identified multiple homologues of the motif, most of which belong to MATE family efflux transporters, ABC transporter permeases, and M56 family metallopeptidases. To ensure reproducibility, the modeling procedure was independently repeated three times for each sequence, and consistent template selection and model quality metrics were obtained across runs.

To explore whether the amyloidogenic motif (SIAIILCILIL) is conserved in bacterial proteins, I searched Gram-negative and Gram-positive bacterial sequences in the NCBI database [29]. Interestingly, no homologs were detected in Gram-negative species. In contrast, multiple proteins from Gram-positive Firmicutes contained this sequence (Figure S1). Notably, four proteins in *C. difficile* harbored this amyloidogenic motif: one was identified as a DUF4116 domain-containing protein, while the others were hypothetical proteins (Figure 2). Further analysis revealed that this amyloidogenic motif was recurrently present in members of the MATE family of efflux transporters, ABC transporter permeases, and the M56 family of metallopeptidases in *C. difficile* (Figure 3). Motif frequency analysis indicated that the SIAIILCILIL sequence occurs at relatively low frequency in bacterial proteomes.

These computational findings suggest that several *C. difficile* proteins contain sequence motifs with predicted aggregation propensity. To further explore whether *C. difficile* produces extracellular structures capable of interacting with Congo Red, Congo Red-supplemented agar assays were performed.

### 3.4. Experimental Evidence of Congo Red-Binding Structures in C. difficile

**Figure 4:**
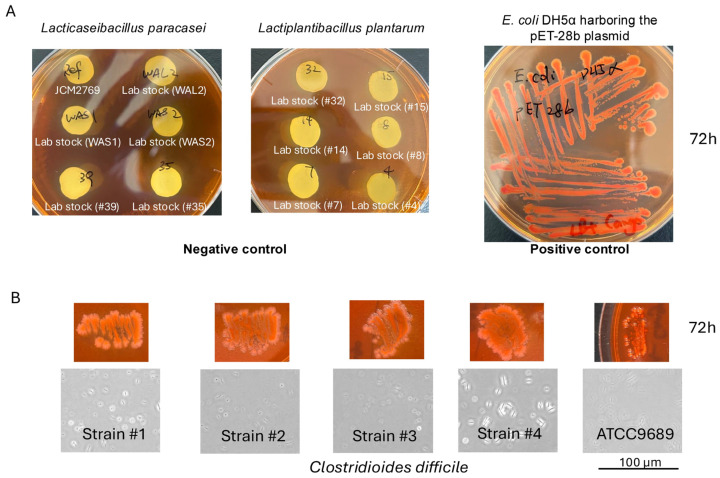
Congo Red binding phenotype of *C. difficile* isolates (**A**) Negative controls included six isolates of *Lacticaseibacillus paracasei* and six isolates of *Lactiplantibacillus plantarum*, which showed no detectable Congo Red binding under the tested conditions. As a positive control, *Escherichia coli* DH5α harboring the pET-28b plasmid was included. All experiments were performed in at least three independent biological replicates to ensure reproducibility. (**B**) Five *C. difficile* isolates developed a distinct red colony phenotype after 48 h of growth on GAM agar supplemented with 1% (*w*/*v*) Congo Red, indicating the presence of Congo Red-binding extracellular components in this species. All experiments were performed in at least three independent biological replicates to ensure reproducibility.

Based on our in silico predictions of amyloidogenic sequences, I hypothesized that *C. difficile* produces amyloid-like proteins. To test this, I performed a Congo Red-agar assay. Congo Red is a dye historically used in amyloid research because of its ability to interact with certain β-sheet-rich structures, leading to red pigmentation of colonies that retain the dye. Appropriate negative and positive controls were included to validate the Congo Red assay (Figure 4A). Negative control strains (*Lacticaseibacillus paracasei* and *Lactiplantibacillus plantarum*) showed no detectable binding, whereas the positive control strain (*E. coli* DH5α harboring pET-28b) exhibited clear Congo Red retention under identical conditions. After 48 h of growth on GAM agar supplemented with 1% (*w*/*v*) Congo Red, both the clinical isolates and the laboratory reference strain exhibited a distinct red colony phenotype (Figure 4). These findings indicate the presence of Congo Red-binding extracellular structures in *C. difficile*. However, because Congo Red binding is not specific to amyloid fibrils, this observation cannot be attributed to the predicted motif or to specific proteins identified in the computational analysis (Table 4). However, because Congo Red binding is not specific to canonical amyloid fibrils, these observations should be regarded as supportive but not conclusive evidence of amyloid formation in this species.

## 4. Discussion

In this study, I performed a detailed analysis of mammalian SAA structures and identified a conserved 19-amino acid sequence that can be useful for predicting the evolutionary relationships among SAA isoforms. In silico predictions revealed that murine SAA3 exhibits the strongest aggregation propensity. Sequence analysis of murine SAA3 identified an amyloidogenic motif (SIAIILCILIL), which was also found to be widely present in *C. difficile*. This approach, which leverages the sequence features of mammalian amyloid proteins to identify potential amyloid-like proteins in bacteria, provides a framework for detecting motifs broadly conserved across mammals and bacteria, thereby advancing our understanding of the physiological and pathological roles of amyloid proteins.

Several well-characterized bacterial amyloids, including those formed by CsgA in *Escherichia coli*, TasA in *Bacillus subtilis*, FapC in *Pseudomonas species*, and phenol-soluble modulins (PSMs) in *Staphylococcus aureus*, are encoded by sequences intrinsically enriched in β-sheet-favoring motifs and form amyloid fibrils through well-defined cross-β architectures [13]. In these systems, amyloidogenic propensity is largely embedded within primary sequence determinants that predispose the polypeptide toward β-aggregation. Notably, while previous studies have primarily focused on intrinsically β-sheet-rich bacterial amyloids, our findings suggest that aggregation-prone motifs may also arise from α-helical regions, indicating a potential underexplored mechanism.

In contrast, the SIAIILCILIL motif identified in this study emerges from a region predicted to adopt α-helical structure, suggesting a distinct structural context. Rather than representing a constitutively β-prone segment, this motif may possess latent aggregation potential that becomes apparent through α-helix-to-β-sheet conformational transition. This conceptual shift—from detecting intrinsically β-rich sequences to identifying helix-derived amyloidogenic candidates—highlights a complementary discovery strategy that expands beyond traditional β-centric paradigms of bacterial amyloid identification. By focusing on motifs embedded within α-helical regions, our approach may facilitate systematic exploration of proteins capable of structural plasticity-driven amyloidogenesis, thereby broadening the current framework of bacterial amyloid research.

By applying this cross-species, sequence-based strategy, I provide preliminary observations suggesting the presence of Congo Red-binding extracellular structures in *C. difficile*. These observations suggest that sequence motifs with similar physicochemical properties may occur in both mammalian and bacterial proteins. Although a potential association between *C. difficile* infection and AA amyloidosis has been reported, current evidence remains limited and is primarily based on isolated clinical observations [30]. Therefore, the proposed mechanistic link should be interpreted with caution. Our findings raise the possibility that persistent inflammatory responses during *C. difficile* infection could contribute to conditions favorable for AA amyloid deposition; however, a direct causal relationship has not been established and warrants further investigation.

## 5. Limitations

The present study has several limitations. However, the lack of direct biochemical validation highlights a gap between computational prediction and experimental confirmation in current amyloid research. First, our experimental validation relied primarily on Congo Red-binding assays. While widely used as a proxy for amyloid-like structures, this method does not provide definitive evidence of fibril formation or identify the specific protein species involved. In bacterial systems, Congo Red may also interact with extracellular polysaccharides, surface-associated proteins, or cell wall components, potentially confounding interpretation. Therefore, Congo Red-binding alone cannot be considered definitive evidence of amyloid formation [31,32]. Second, the analysis was restricted to *C. difficile*, and it remains uncertain whether analogous motifs in other bacterial species form amyloids or contribute to their physiological processes. Third, although our computational predictions indicate strong amyloidogenic potential, further biophysical investigations (such as Thioflavin T fluorescence assays, transmission electron microscopy, or solid-state NMR) are required to conclusively demonstrate fibril formation and define the precise molecular entities responsible.

The identification of similar amyloid-like motifs in both mammalian and bacterial proteins raises intriguing evolutionary questions. Such similarities may reflect convergent evolution driven by shared physicochemical constraints that favor aggregation-prone sequence patterns, particularly hydrophobic residues capable of structural transition. Alternatively, it is possible that certain aggregation-prone motifs represent deeply conserved sequence features retained for functional or structural reasons.

The present study explored whether conserved sequence features derived from mammalian amyloid-associated proteins could be used to identify candidate aggregation-prone motifs in bacterial proteomes. Through comparative sequence analysis and in silico aggregation prediction, a hydrophobic motif (SIAIILCILIL) located within murine SAA3 was identified as a segment with predicted aggregation propensity. Database searches further revealed that similar sequence patterns occur in several proteins encoded by *C. difficile* and other Gram-positive bacteria. These observations suggest that bacterial proteomes may contain sequence elements with physicochemical characteristics compatible with amyloid-like aggregation.

However, the current study provides primarily computational evidence, and the experimental observations presented here do not establish a direct causal link between the identified motif and the Congo Red-binding phenotype observed in *C. difficile* colonies. Congo Red binding can arise from several extracellular components, including polysaccharides, surface-associated proteins, or other matrix constituents [31,32]. Therefore, the Congo Red assay used in this study should be interpreted as a phenotypic observation rather than a direct demonstration of amyloid formation by the predicted motif or by specific proteins identified through the bioinformatic analysis.

Consequently, the results of this study should be regarded primarily as a hypothesis-generating analysis highlighting candidate sequence motifs that may warrant further investigation.

## 6. Future Perspectives

Future studies should focus on employing biochemical and structural approaches—such as peptide aggregation assays, transmission electron microscopy, circular dichroism spectroscopy, or solid-state structural methods—which will be required to determine whether these predicted motifs indeed form amyloid-like assemblies and to identify the specific protein species involved.

## 7. Conclusions

Despite these limitations, the motif-based framework presented here may provide a complementary strategy for exploring aggregation-prone sequence patterns across species. Rather than relying exclusively on homology to known bacterial amyloid proteins, this approach focuses on conserved physicochemical features that may enable structural transitions associated with aggregation. Such strategies may help broaden the search space for previously unrecognized amyloid-like proteins in microbial proteomes and generate new hypotheses regarding possible structural convergence between host and microbial amyloid systems. Therefore, the present work should be regarded primarily as a hypothesis-generating computational study highlighting candidate aggregation-prone motifs for future experimental validation.

## Figures and Tables

**Table 1:** Sequence of mammalian SAA isoforms.

SAA Isoforms	UniProt ID	Sequence (from N-Terminus to C-Terminus)	Length (AAs)	Position
Equus caballus (Horse)-SAA	P19857 · SAA_HORSE	MKKPLVSARKPDYKYQPPLPRGRDRQLCSPEVSTMKLFTGLVFCSLVLGASGLLSFLGEAARGTWDMLRAYHDMREANYIGADKYFH**ARGNYDAAQRGPGGAWAAK**VIRSPAERDSHSKMKLSIGIIFCSLVLGVSSREWFTFLKEAGQGAKDMWRAYSDMREANYIGADKYFH**ARGNYDAAQRGPGGAWAAK**VISDARENAQRFTDRFKFGDSGQGAADSRADQAANEWGRSGKDPNHFRPRGLPDKY	249	88–106; 175–193
Sus scrofa (Pig)-SAA3	A0A5G2QFI9 · A0A5G2QFI9_PIG	MCGIYFGNARMGQHSPKHPIPFILGCVCSSLCSRMKLSTGIIFCFLILGVSSQRWASFLKEAGQGAKDMWRAYSDMREANYKNSDKYFH**ARGNYDAAQRGPGGAWAAK**VISDARENVQRVTDLFKHGDSGHGVEDSRADQAANAWGRSGKDPNHFRPRGLPDKY	164	90–108
Sus scrofa (Pig)-SAA2	F1S9C0 · F1S9C0_PIG	MKLFTGLIFCSLVLGVHSQWLSFLGEAYEGKTHGGDMAPVAYSDMREANFKNSDKYFH**ARGNYDAAQRGPGGAWAAK**VISDARENVQRVTDWLKHGDSGHGVEDSRADQAANEWGRSGKDPNHFRPPGLPDKY	133	58–76
Bos taurus (Cattle)-SAA1	P35541 · SAA_BOVIN	MKLFTGLILCSLVLGVHSQWMSFFGEAYEGAKDMWRAYSDMREANYKGADKYFH**ARGNYDAAQRGPGGAWAAK**VISDARENIQRFTDPLFKGTTSGQGQEDSRADQAANEWGRSGKDPNHFRPAGLPDKY	130	55–73
Bos taurus (Cattle)-SAA3	Q8SQ28 · SAA3_BOVIN	MNLSTGIIFCFLILGVSSQRWGTFLKEAGQGAKDMWRAYQDMKEANYRGADKYFH**ARGNYDAARRGPGGAWAAK**VISNARETIQGITDPLFKGMTRDQVREDSKADQFANEWGRSGKDPNHFRPAGLPDKY	131	56–74
Mus musculus (Mouse)-SAA1	P05366 · SAA1_MOUSE	MKLLTSLVFCSLLLGVCHGGFFSFVHEAFQGAGDMWRAYTDMKEANWKNSDKYFH**ARGNYDAAQRGPGGVWAAEK**ISDGREAFQEFFGRGHEDTIADQEANRHGRSGKDPNYYRPPGLPDKY	122	56–74
Mus musculus (Mouse)-SAA2	P05367 · SAA2_MOUSE	MKLLTSLVFCSLLLGVCHGGFFSFIGEAFQGAGDMWRAYTDMKEAGWKDGDKYFH**ARGNYDAAQRGPGGVWAAEK**ISDARESFQEFFGRGHEDTMADQEANRHGRSGKDPNYYRPPGLPAKY	122	56–74
Mus musculus (Mouse)-SAA3	P04918 · SAA3_MOUSE	MKPSIAIILCILILGVDSQRWVQFMKEAGQGSRDMWRAYSDMKKANWKNSDKYFH**ARGNYDAARRGPGGAWAAK**VISDAREAVQKFTGHGAEDSRADQFANEWGRSGKDPNHFRPAGLPKRY	122	56–74
Homo sapiens (Human)-SAA1	P0DJI8 · SAA1_HUMAN	MKLLTGLVFCSLVLGVSSRSFFSFLGEAFDGARDMWRAYSDMREANYIGSDKYFH**ARGNYDAAKRGPGGAWAAE**VITDARENIQRFFGHGAEDSLADQAANEWGRSGKDPNHFRPAGLPEKY	122	56–74
Homo sapiens (Human)-SAA2	P0DJI9 · SAA2_HUMAN	MKLLTGLVFCSLVLSVSSRSFFSFLGEAFDGARDMWRAYSDMREANYIGSDKYFH**ARGNYDAAKRGPGGAWAAE**VISNARENIQRLTGRGAEDSLADQAANKWGRSGRDPNHFRPAGLPEKY	122	56–74
Homo sapiens (Human)-SAA4	P35542 · SAA4_HUMAN	MRLFTGIVFCSLVMGVTSESWRSFFKEALQGVGDMGRAYWDIMISNHQNSNRYLY**ARGNYDAAQRGPGGVWAAK**LISRSRVYLQGLIDCYLFGNSSTVLEDSKSNEKAEEWGRSGKDPDRFRPDGLPKKY	130	56–74

**Table 2:** Predicted aggregation propensity of mammalian SAA isoforms.

Protein Name	Length (AAs)	Best Energy	% Disorder	% α-Helix	% β-Strand	% Coil
Mouse SAA3	122	−12.372043	31.96	63.93	0	36.07
Cattle SAA3	131	−9.949253	29.77	67.18	0	32.82
Pig SAA3	164	−9.949253	28.04	59.76	1.83	38.41
Horse SAA	249	−9.154135	30.12	61.85	0	38.15
Human SAA4	130	−8.351752	30.76	43.85	12.31	43.85
Mouse SAA1	122	−8.246094	35.24	64.75	0	35.25
Mouse SAA2	122	−8.214458	32.78	62.3	0	37.7
Human SAA2	122	−8.169815	31.96	69.67	0	30.33
Pig SAA2	133	−8.05708	36.84	59.4	0	40.6
Human SAA1	122	−7.500759	27.86	68.85	0	31.15
Cattle SAA1	130	−7.478989	43.84	66.92	0	33.08

**Table 3:** Predicted aggregation propensity of different sequences in murine SAA3.

Protein Name	Length (AAs)	Sequence	Best Energy	% Disorder	% α-Helix	% β-Strand	% Coil
whole mouse SAA3	122	MKPSIAIILCILILGVDSQRWVQFMKEAGQGSRDMWRAYSDMKKANWKNSDKYFHARGNYDAARRGPGGAWAAKVISDAREAVQKFTGHGAEDSRADQFANEWGRSGKDPNHFRPAGLPKRY	−12.37204	31.96	63.93	0	36.07
hypothetical amyloidogenic motif	11	SIAIILCILIL	−11.70283	54.54	0	63.64	36.36
mouse SAA3 (deletion of amyloidogenic motif)	108	GVDSQRWVQFMKEAGQGSRDMWRAYSDMKKANWKNSDKYFHARGNYDAARRGPGGAWAAKVISDAREAVQKFTGHGAEDSRADQFANEWGRSGKDPNHFRPAGLPKRY	−2.539415	34.25	64.81	0	35.19

**Table 4:** Comparison of amyloid-related features between mammalian SAA and *C. difficile* proteins.

Feature	Mammalian SAA	*C. difficile* Proteins
Motif	SIAIILCILIL	Present
Structure	α-helical	Unknown
Aggregation (PASTA 2.0)	High (SAA3)	Predicted
Experimental evidence	No	Congo Red binding

This table summarizes key structural, computational, and experimental features supporting the cross-species identification of candidate amyloid-like motifs.

## Data Availability

The original contributions presented in this study are included in the article/Supplementary Material. Further inquiries can be directed to the corresponding author.

## Supplementary Materials

The following supporting information can be downloaded at: https://www.mdpi.com/article/10.3390/microorganisms14040821/s1, Figure S1: BLAST searches of *E. coli* CsgA in Gram-positive and Gram-negative bacteria. (A) BLAST search results using *E. coli* CsgA (UniProt ID: P28307, CSGA_ECOLI) in Gram-positive bacteria (taxid:1239). (B) BLAST search results using the same query sequence in Gram-negative bacterium 0471 (taxid:204774). The alignments illustrate the distribution and conservation of CsgA-like sequences across distinct bacterial taxa.
