# Supplementary material for: Cross-Species Amyloid-like Features Shared by Mammalian and Clostridioides difficile Proteins
Source: Microorganisms. 2026 Apr 2;14(4):821. doi: 10.3390/microorganisms14040821 (PMC13119156; doi:10.3390/microorganisms14040821)
Supplement: Supplementary file 1 [file microorganisms-14-00821-s001.zip › microorganisms-4228943-supplementary.pdf]

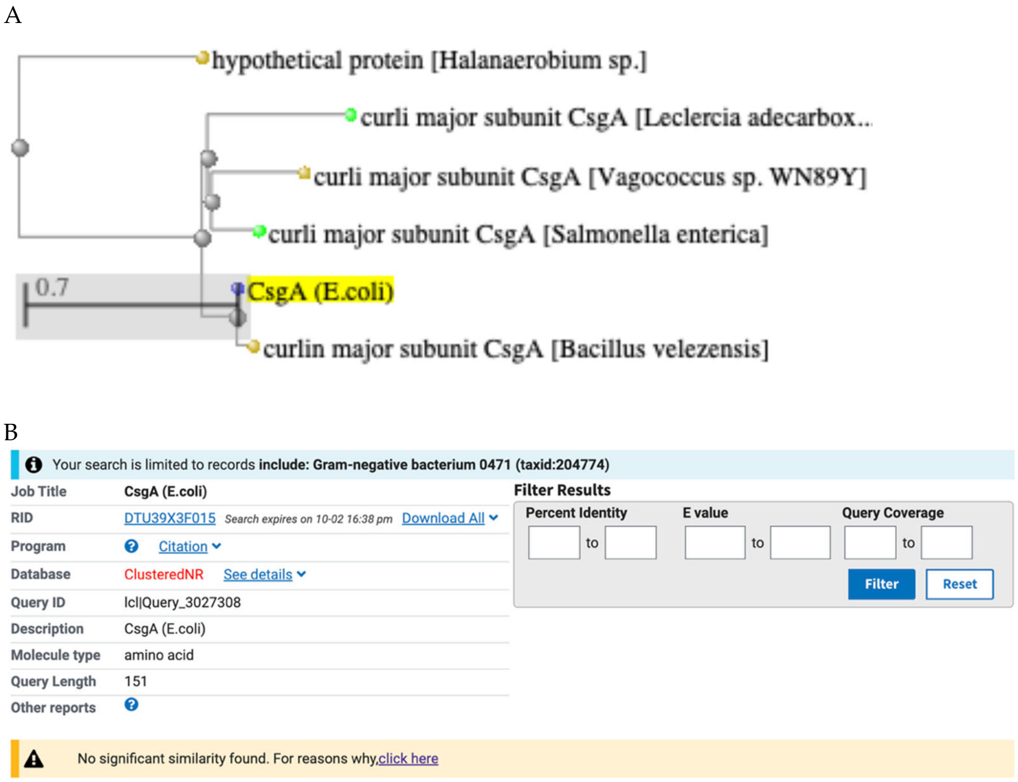

Figure S1. BLAST searches of *E. coli* CsgA in Gram-positive and Gram-negative bacteria

(A) BLAST search results using *E. coli* CsgA (UniProt ID: P28307, CSGA_ECOLI) in Gram-positive bacteria (taxid:1239). (B) BLAST search results using the same query sequence in Gram-negative bacterium 0471 (taxid:204774). The alignments illustrate the distribution and conservation of CsgA-like sequences across distinct bacterial taxa.
